# Lifestyle habits in Saudi adolescents with diagnosed diabetes: An opportunity for health promotion

**DOI:** 10.1371/journal.pone.0270807

**Published:** 2022-08-04

**Authors:** Mona Nasrallah, Hani Tamim, Aurelie Mailhac, Fadia AlBuhairan

**Affiliations:** 1 Department of Internal Medicine, Division of Endocrinology and Metabolism, American University of Beirut Medical Center, Beirut, Lebanon; 2 College of Medicine, Alfaisal University, Riyadh, Saudi Arabia; 3 Department of Internal Medicine, American University of Beirut Medical Center, Beirut, Lebanon; 4 King Abdullah International Medical Research Center, Riyadh, Saudi Arabia; 5 Health Sector Transformation Program, Riyadh, Saudi Arabia; University of Bern, SWITZERLAND

## Abstract

**Aims:**

This study assessed lifestyle and health behavior habits among a representative sample of Saudi adolescents with self-reported diabetes and compared them to non-diabetic peers.

**Methods:**

This was a nested case-control study, from the Jeeluna cohort, a nationwide, cross-sectional study of 12,575 Saudi boys and girls aged 10–19 years. Non-diabetic adolescents were matched to those with diabetes on a ratio of 4:1 based on age, gender and region. Retained information from the original study included: socio-demographics, lifestyle behaviors, tobacco/substance use, screen use, anthropometric measurements, and laboratory results.

**Results:**

The prevalence of diabetes was 0.7% (n = 87). Overall, 65% of diabetic participants were males, and 22.4% aged ≤14 years. Overall, both groups had low rates of healthful habits in their diet and physical activity. Both groups had similar rates of tobacco use, and high digital screen time. Adolescents with diabetes had more consistent sleeping pattern, were more likely to be on a diet, thought they spent enough time with their physician and obtained medical information more often from their health clinic. They were also more likely to feel down and to chat more often.

**Conclusion:**

Adolescents with diabetes remain far from guideline targets but seem predisposed to better lifestyle and have more access to health as compared to their non-diabetic peers.

## Introduction

Both Type 1 and 2 diabetes are on the rise in the adolescent age group [1]. Despite the different underlying pathophysiologic mechanisms and clinical profile, there is commonality in the principles of management between the two types, which include adequate glycemic control and adoption of healthy lifestyle, with the aim of avoiding diabetes’ chronic complications [2].

There is a strong body of evidence associating a healthy lifestyle with better glycemic control and lower complications rate [3, 4], in adolescents with diabetes. The former is clearly in addition to insulin therapy in the case of Type 1 diabetes (T1D), and to other pharmacotherapy when needed in the case of Type 2 diabetes (T2D) [5]. Elements of healthful lifestyle and behavior which have been associated with improved glycemic outcomes include maintenance of a healthy weight and a diet high in fruit/vegetable intake, and low in saturated/transfats and in free sugars [6], regular meal intake [7], moderate to vigorous physical activity of one hour daily [2], adequate and regular sleep of eight to ten hours [8], limited screen time and leisurely media consumption to less than two hours daily [9, 10], and finally refraining from any kind of smoking including e-cigarettes [2].

Adolescence is a time period documented to have worsening of glycemic control [11]. Part of the deterioration may be physiologic such as increased insulin requirements, and the other part is largely due to decreased adherence to treatment and to healthy habits [12]. The physiologically increased insulin requirements are related to the increase in growth hormone and sex steroids in puberty. Adolescents with diabetes may be influenced by peers, and additionally have to face the increasing challenge of self-care of a chronic condition. Consequently, these factors may affect the compliance to a healthy routine, during this phase of their life. Furthermore, lifestyle is very much culture and generation-dependent, and may vary considerably when comparing one group of adolescents to another, from a different time and place [13].

The Middle-East North Africa (MENA) region is reported to have high prevalence of diabetes in general, and one of the highest rates of rise of T2D [14]. Even though national studies are scarce, there is evidence that the adolescent age group is not spared from this epidemic with high levels reported for both T1D [15] and T2D [14]. The MENA region comprises most of the Arab countries, and despite some heterogeneity in terms of economic level, population density, and national health systems, adolescents from this region do share some commonalities in their culture, religion, and geography, which in turn may influence their health habits [16].

There exist very few studies describing the lifestyle and behavior of adolescents from the MENA region [17]. Furthermore, there exist only few studies in general comparing the lifestyle of adolescents with diabetes to those without, and none from the MENA region [18]. Despite the relatively rich body of evidence linking healthful lifestyle in diabetes mellitus to improved outcomes, the paucity of data and the variation in lifestyle and health habits between populations and over time calls for more studies from different regions. Moreover, in order to promote the American Diabetes Association recommendations for healthy lifestyle, it would be important to assess where adolescents with diabetes from the MENA region stand, and to compare them to their peers without diabetes, from a similar background and culture.

Thus, the primary aim of this study was to identify factors associated with diabetes, mainly, lifestyle habits and health behaviors, among Saudi adolescents with diagnosed diabetes and compare them to non-diabetic peers.

## Methods

### Original study

The current study is a secondary analysis of the ‘JEELUNA’ study, which was a school-based national adolescent health cross-sectional survey conducted in 2011–2012 in the Kingdom of Saudi Arabia (KSA) [17]. Its main objective was to evaluate the health needs and status of adolescents in the KSA. Details of the original study have been published elsewhere [17]. Briefly, a representative sample of 12,575 adolescents, mean age 15.8 ±3.4 years, 51% boys, and from the 13 different regions of KSA was selected from schools using a stratified, cluster, random sampling procedure. Respondents who agreed to participate in the study filled up a self-administered questionnaire that gathered information on sociodemographic, lifestyle, nutrition, physical activity, sleep, smoking and substance use, as well as past medical history. Moreover, anthropometric data and laboratory tests were obtained. The student response rate was 32.7%. Institutional review board (IRB) approval was obtained from the King Abdullah International Medical Research Center (KAIMRC). Moreover, parents provided consent for the participation of their children, as well an assent was obtained from the participants.

### Current study

The current study had a nested case-control design within the participants of the Jeeluna study. All participants aged between 11 and 18 years old were included. Individuals with missing information on diabetes status (whether being diabetic or not) were excluded from this study. Because the current study is a secondary data analysis, the sample size was determined by the original study of 12,575 adolescents based on the objectives of that study. Out of that sample, all participants who qualify as ‘cases’ were included and matched with 4 ‘controls’ for efficiency.

### Cases and controls

Cases were defined as all participants who responded “Yes” to the following question: “have you been diagnosed with Diabetes”, and accordingly considered as having diabetes. On the other hand, eligible controls were those who answered “No” to the above question, and thus, considered not having diabetes. For matching, 4 controls per case were randomly selected from the pool of eligible controls based on 3 matching criteria: age (exact age), gender, and region. Despite being one country, there exists some differences in culture and lifestyle among inhabitants of the various regions, and so this variable was included in the matching criteria. Accordingly, participants with missing information on any of these three matching variables were not considered in the pool of eligible cases or controls.

### Data used

Data available in the original database included information from a self-administered questionnaire, anthropometric measurements, and laboratory studies. The self-administered questionnaire aimed to capture information on different domains pertinent to adolescents’ health. Following is a description of each of these domains that are used in this study.

#### Socio-demographic and school-related information

We retained from the database information on demographics (age, gender, and grade), socioeconomic status (paternal and maternal level of education, monthly family income), and school-performance related information (average academic performance in the prior semester, frequency and reason of school absenteeism, and having ever been dismissed/expelled from school).

#### Lifestyle

Participants’ dietary habits were retained from the database, specifically relating to intake of breakfast, main meals, snacks, fruits, vegetables, soft drinks, power drinks, milk, fast food, and meal skipping. We computed a dietary score for each individual by adding up the number of sub-optimal behaviors the adolescent reports. More specifically, suboptimal behaviors were given a score of 1, and were considered for consumption of breakfast (irregular), main meal (<3 meals/day), fruits (<2 fruits/day), vegetables (<2 times/day), soft drinks (one or more/day), power drinks (one or more/day), milk (none or irregular), fast food (≥2 days/week), and skipping of meals (in the last 7 days). The score ranged from 0 to 9 according to the number of suboptimal behaviors. It was further categorized into ≤3 and >3 to reflect on the appropriateness of the eating behavior. In addition, we reported the number of snacks the adolescent had per day and categorized it as <2 and ≥2. As for physical activity, it was assessed by asking about the number of times they exercised for 30 minutes/day in the last 7 days. Finally, sleep patterns were assessed by reporting the number of hours of sleep the adolescent usually had during the weekdays and weekends separately (<8, 8 to 10, and >10 hours) [8].

#### Tobacco/Substance use

Information retained from the database pertaining to tobacco/substance use included information on any use of cigarette, shisha (hookah/waterpipe), alcohol, marijuana, solvent sniffing, and illegal pills. All these elements were integrated into a substance use score. The score counted how many of those items the adolescent had ever tried and consequently ranged from 0 to 6.

#### Screen use

Screen use was assessed by asking questions about the average daily use of TV, video games, internet use for non-school related issues, mobile phone, and chatting on internet. An electronics consumption score was created by adding up how many suboptimal behaviors out of 4 elements the participant had. The 4 suboptimal behaviors for the score were considered as spending ≥2 hours/day on TV, more than 2 hours/day on the internet for non-school related issues, more than 2 hours/day on video games, or more than 2 hours/day on the mobile phone. The score accordingly ranged from 0 to 4. The screen time cutoffs for classification into suboptimal behavior were chosen according to the American Academy of Pediatrics’ recommendations [9].

#### Anthropometric measurements

Anthropometric measures (such as weight, height, systolic and diastolic blood pressure, and waist circumference) were taken with students in light clothes and without shoes. Height was measured using a height chart on the wall to the nearest 0.5 cm. Weight was measured using a calibrated electronic scale to the nearest 0.1 kg (OmronSC100 digital scale, USA). Each participant body mass index (BMI) was classified according to the Center for Disease Control BMI charts and as follows [19]: underweight (<5^th^ percentile), healthy weight (between 5^th^ to <85^th^ percentile), overweight (between 85^th^ to <95^th^ percentile), or obese (≥95^th^ percentile). Waist circumference was measured with a non-elastic tape. The measurement was taken at the end of expiration, at the midpoint between the coastal margin and iliac crest. It was noted to the nearest mm. Systolic and diastolic blood pressures (BP) were measured using a digital BP monitor (Omron M2, Netherlands) placed on the right arm. The average of two measurements was considered in this analysis.

#### Other health-related information

Participants were asked about their perception of their body weight, and if they were currently on diet. Moreover, participants were asked whether they have been diagnosed with any chronic disease. Furthermore, the mental health status was evaluated through 3 questions regarding the following symptoms over the past year (as published in a previous paper using the same database): ever felt sad or down for more than 2 weeks, ever sought help for feeling down, and ever felt excessive worry that affected the participant’s daily activities [20]. Access to healthcare was evaluated by asking the participants if they felt it was easy to access health care when needed, about the last time they saw a doctor, and whether they thought they spent enough time with the physician during their last consultation. Access to health information was evaluated by asking where they obtain their medical information from (school, doctor/nurse, family, TV, internet, newspaper, or friends).

#### Laboratory results

Laboratory results available in the database included: fasting glucose level, lipid profile (cholesterol, triglycerides, HDL, and LDL), hemoglobin, hematocrit, ferritin, calcium, and total vitamin D. Blood samples were collected on a subset of the recruited participants. The concerned students were required to have fasted for the last 8 hours. Collected samples were transported within 24 hours of withdrawal to the King Abdulaziz Medical City in Riyadh where they were analyzed for the above measures. Cell counter hematology analyzer (Abbott, USA), centrifugation for 10–15 min at 3000 RPM with separation and storage at -70 degrees Celsius and automated chemistry analyzer Architect 16000 (Abbott, USA) were the methods used for analyses.

### Statistical analyses

Descriptive statistics were performed by diabetic status. Frequencies and percent were used for categorical variables whereas means and standard deviation (SD) were used for continuous ones. Characteristics were then compared between diabetic and non-diabetic participants. The chi-square and t-test were used for the categorical and continuous variables, respectively. Moreover, multivariate logistic regression was used to identify lifestyle associated factors with diabetes status. The variables included in this regression analysis were: eating pattern, physical activity, sleep pattern, body mass index, substance use, and electronics use. Results were reported as Odds Ratio (OR) and 95% Confidence Interval (CI). P-values were two-sided and an alpha of 5% was set for statistical significance. All analyses were conducted using SAS version 9.4.

## Results

Out of a total of 12,575 students (the total Jeeluna cohort), 11,983 had answered the question about diabetes. Out of these, 87 (prevalence of 0.7%, 95% CI: 0.5–0.8) participants answered yes, and 11896 (99.3%) answered no. Out of the diabetic adolescents, 76 were aged between 11 and 18 years. Therefore, the final sample of diabetic adolescents included in the study was 76 (cases), to which 304 non-diabetic (controls) were matched.

A socio-demographic and school-related description and comparison between cases and controls is presented in Table 1. Mainly, among cases, 17 (22.4%) were aged ≤14 years and 50 (65.8%) were males. No statistically significant difference was observed between cases and controls on the other variables. In general, about one third of our sample came from a lower socioeconomic status as classified by income of <5000 SR (equivalent to 1,300 US$), and by parents’ education reaching primary school level only. Self-reported school performance was good or above for the majority of our sample (68.5% and 75.9%, for cases and controls respectively).

**Table 1:** Socio-demographic and school-related information for the participants in the study.

		Cases	Controls	
		(n = 76)	(n = 304)	p-value
**Demographics**				
Age, years	≤14	17 (22.4)	68 (22.4)	1.00
	>14	59 (77.6)	236 (77.6)	
Gender	Male	50 (65.8)	200 (65.8)	1.00
	Female	26 (34.2)	104 (34.2)	
Grade	Intermediate	41 (54.0)	160 (52.6)	0.84
	Secondary	35 (46.1)	144 (47.4)	
**Socioeconomic status**				
Father’s level of education	Up to primary	19 (27.1)	65 (25.1)	0.60
	Middle or high school	27 (38.6)	117 (45.2)	
	College and above	24 (34.3)	77 (29.7)	
Mother’s level of education	Up to primary	25 (36.8)	99 (37.9)	0.98
	Middle or high school	25 (36.8)	95 (36.4)	
	College and above	18 (26.5)	67 (25.7)	
Monthly income for family	< 5000 SR	14 (33.3)	38 (23.6)	0.44
	5000–15000 SR	18 (42.9)	79 (49.1)	
	≥ 15 000 SR	10 (23.8)	44 (27.3)	
**School performance**				
Previous GPA (prior semester)	Very weak or failed	4 (5.5)	9 (3.1)	0.37
	Average	19 (26.0)	61 (21.0)	
	Good and above	50 (68.5)	220 (75.9)	
Frequency of being absent*	Frequent	4 (5.3)	8 (2.7)	0.27
	Infrequent	71 (94.7)	286 (97.3)	
Reason for being absent	Medical	54 (88.5)	196 (80.0)	0.12
	Non-medical	7 (11.5)	49 (20.0)	
Ever been dismissed from school	Yes	10 (14.1)	27 (9.2)	0.22
	No	61 (85.9)	267 (90.8)	

*: infrequent was defined as never, rarely, or sometimes, whereas frequent was defined as often or always

Results of the comparison of lifestyle characteristics between cases and controls, mainly dietary habits, physical activity, and sleep patterns, are presented in Table 2. There were no significant differences in lifestyle habits according to the above-mentioned characteristics between cases and controls, with only about a quarter of the participants leading a healthier lifestyle as reflected by the overall score of ≤3 for 26.3% and 20.1% of cases and controls, respectively. As an example, consuming ≥ 2 fruits per day was limited to 24.3% and 19.8%, for cases and controls, respectively, and ≥2 vegetables per day to 29.7% and 20.9%, for cases and controls, respectively. Both groups had a high rate of daily soft drink intake, and low rates of physical activity, with only 43% of cases and 30% of controls exercising for 30 minutes more than twice per week. With regards to sleep habits, cases were more likely to sleep more than 10 hours during weekdays as compared to controls (18.1% versus 7.8%, p-value = 0.01), whereas both groups had a proportion of around 20% sleep more than 10 hours during weekends.

**Table 2:** Lifestyle characteristics of study participants, mainly dietary habits, physical activity, and sleep hours.

		Cases	Controls	
		(n = 76)	(n = 304)	p-value
**Dietary habits**				
Regularly having breakfast	No	43 (57.3)	194 (65.8)	0.17
	Yes	32 (42.7)	101 (34.2)	
Number of main meals per day	< 3	29 (38.7)	131 (43.7)	0.49
	3	29 (38.7)	118 (39.3)	
	> 3	17 (22.7)	51 (17.0)	
Skipping meals in the last 7 days	Yes	44 (59.5)	177 (61.3)	0.78
	No	30 (40.5)	112 (38.8)	
Number of snacks per day	< 2	34 (45.3)	142 (47.3)	0.76
	≥ 2	41 (54.7)	158 (52.7)	
Number of fruits per day	< 2	56 (75.7)	243 (80.2)	0.39
	≥ 2	18 (24.3)	60 (19.8)	
Frequency of vegetable consumption per day	< 2	52 (70.3)	238 (79.1)	0.10
	≥ 2	22 (29.7)	63 (20.9)	
Frequency of soft drink consumption per day	One or more	49 (66.2)	185 (61.5)	0.45
	None or irregular	25 (33.8)	116 (38.5)	
Frequency of power drink consumption per day	One or more	14 (18.9)	74 (24.6)	0.30
	None or irregular	60 (81.1)	227 (75.4)	
Number of cups milk per day	None or irregular	34 (46.0)	163 (55.1)	0.16
	One or more	40 (54.1)	133 (44.9)	
Number of days with fast food consumption in the last 7 days	> 3	14 (18.9)	58 (19.6)	0.53
	2–3	29 (39.2)	96 (32.4)	
	<2	31 (41.9)	142 (48.0)	
Score for eating behavior*	>3	56 (73.7)	243 (79.9)	0.23
	≤3	20 (26.3)	61 (20.1)	
**Physical activity**				
How many times in the last 7 days did you do exercise for 30 min	Never	25 (33.8)	131 (44.3)	0.09
	1–2	17 (23.0)	76 (25.7)	
	˃ 2	32 (43.2)	89 (30.1)	
**Sleep patterns**				
Number of hours sleep during the weekdays	< 8	43 (59.7)	170 (57.8)	0.01
	8–10	16 (22.2)	101 (34.4)	
	> 10	13 (18.1)	23 (7.8)	
Number of hours sleep during the week ends	< 8	33 (45.8)	125 (42.2)	0.85
	8–10	25 (34.7)	111 (37.5)	
	> 10	14 (19.4)	60 (20.3)	

*Original score created by adding up the number of the 9 following suboptimal behaviors consumptions of breakfast (irregular), main meal (<3 meals/day), fruit (<2 fruits/day), vegetable (<2 times/day), soft drink (one or more/day), power drink (one or more/day), milk (one or more cups/day), fast food (≥2 days/week), and skipping of meals (in the last 7 days)

Results regarding anthropometrics, mental health and access to healthcare and health knowledge, as well as laboratory results are presented in Table 3. Around one third of our sample were overweight or obese. Although there was no significant association between DM status and obesity indicators such as BMI or waist circumference, more cases reported being on a diet as compared to controls (25.0% versus 12.7%, p-value = 0.009). The percentage of adolescents feeling sad or down during the past 12 months were 64.9% and 53.6%, for cases and controls, respectively; and cases were more likely to seek help for it (23.4% versus 11.6%, p-value = 0.03). As for blood tests, cases had significantly higher levels of fasting serum glucose (10.17±8.44 versus 4.50±0.68 mmol/L, p-value<0.001), triglycerides (1.19±0.82 versus 0.86±0.38 mmol/L, p-value = 0.03) and LDL cholesterol (2.72±0.82 versus 2.36±0.65 mmol/L, p-value = 0.005) as compared to controls. They were also more likely to have seen a physician within the last 3 months (78.4% versus 61.9%, p-value = 0.008), and to report spending enough time with their physician (72.9% versus 54.2%, p-value = 0.02).

**Table 3:** Anthropometric measurements, health-related information, and laboratory results pertaining to study participants.

			Cases	Controls	
			(n = 76)	(n = 304)	p-value
**Anthropometric measurements**					
Weight, kg		Mean ± SD	58.27 ± 16.87	59.99 ± 18.65	0.46
Height, cm		Mean ± SD	160.29 ± 11.17	161.60 ± 9.55	0.30
BMI, kg/m^2^		Mean ± SD	22.54 ± 5.59	22.81 ± 6.08	0.73
SBP, mm Hg		Mean ± SD	121.67 ± 11.46	120.94 ± 11.86	0.66
DBP, mm Hg		Mean ± SD	69.22 ± 8.82	69.53 ± 11.29	0.81
Waist circumference, cm	Males	Mean ± SD	71.19 ± 19.17	73.75 ± 19.46	0.40
	Females	Mean ± SD	70.23 ± 21.84	70.34 ± 16.03	0.98
BMI percentile		<5^th^ percentile	13 (17.1)	43 (14.2)	0.20
		5 - <85^th^ percentile	39 (51.3)	159 (52.7)	
		85 - <95^th^ percentile	14 (18.4)	35 (11.6)	
		≥95^th^ percentile	10 (13.2)	65 (21.5)	
**Weight perception and control**					
Perception about weight		Happy about weight	20 (27.4)	102 (35.1)	0.19
		Need to lose weight	33 (45.2)	135 (46.4)	
		Need to gain weight	20 (27.4)	54 (18.6)	
Whether currently on a diet		No	54 (75.0)	255 (87.3)	0.009
		Yes	18 (25.0)	37 (12.7)	
**Mental health**					
Ever felt sad or down for more than 2 weeks (12 months)		No	26 (35.1)	140 (46.4)	0.08
		Yes	48 (64.9)	162 (53.6)	
Ever sought help for feeling down (12 months)		No	36 (76.6)	176 (88.4)	0.03
		Yes	11 (23.4)	23 (11.6)	
Ever felt worried that affected your daily activities (12 months)		No	40 (54.8)	187 (63.0)	0.20
		Yes	33 (45.2)	110 (37.0)	
Any chronic diseases		No	32 (43.2)	270 (91.8)	<0.001
		Yes	42 (56.8)	24 (8.2)	
**Access to healthcare and health knowledge**					
Is it easy to access health care when needed		No	19 (25.7)	71 (23.8)	0.74
		Yes	55 (74.3)	227 (76.2)	
When was the last time you saw a doctor		≤ 3 months	58 (78.4)	182 (61.9)	0.008
		> 3 months	16 (21.6)	112 (38.1)	
Did you spend enough time with the doctor during your last physician consultation?		No	13 (27.1)	66 (45.8)	0.02
		Yes	35 (72.9)	78 (54.2)	
Where do you get your medical information from?		School	18 (24)	109 (37.1)	0.03
		Doctor/nurse	32 (42.7)	88 (29.9)	0.04
		Family	45 (60.0)	120 (40.8)	0.003
		TV	19 (25.7)	86 (29.3)	0.54
		Internet	23 (30.7)	87 (29.6)	0.86
		Newspaper	7 (9.3)	34 (11.6)	0.58
		Friends	19 (25.7)	55 (18.7)	0.18
**Lab tests**			n = 32	n = 161	
Fasting serum glucose, mmol/L			10.17 ± 8.44	4.50 ± 0.68	<0.001
Calcium mmol/L			2.42 ± 0.09	2.42 ± 0.09	0.82
Cholesterol mmol/L			4.44 ± 1.02	4.01 ± 0.67	0.02
Triglycerides mmol/L			1.19 ± 0.82	0.86 ± 0.38	0.03
HDL mmol/L			1.27 ± 0.30	1.28 ± 0.28	0.88
LDL mmol/L			2.72 ± 0.82	2.36 ± 0.65	0.005
Ferritin ng/mL			54.67 ± 60.66	33.93 ± 25.40	0.06
Hemoglobin g/L			142.47 ± 17.8	142.14 ± 15.52	0.91
Hematocrit (%)			44 ± 5	44 ± 4	0.98
Total Vitamin D nmol/L			28.32 ± 10.22	28.80 ± 11.86	0.86

Table 4 presents results for other lifestyle habits, such as screen use, and substance abuse among the cases and controls. Most of the habits considered in these analyses were not significantly different between cases and controls. Specifically, about two-thirds of both groups spent at least 2 hours per day using electronics for leisurely purposes. However, cases were more likely to chat on the internet as compared to controls (46.0% versus 31.8%, p-value = 0.02). As for the substance abuse, there was no difference between groups and about one third of the group (36.8% of cases and 33.9% of controls), were more likely to have a score 1 or more; indicating ever used at least 1 of the 6 unhealthy or risky behaviors.

**Table 4:** Other lifestyle habits, such as screen use and substance abuse for participants in this study.

		Cases	Controls	
		(n = 76)	(n = 304)	p-value
**Screen use**				
Do you do chatting on internet	No	40 (54.1)	204 (68.2)	0.02
	Yes	34 (46.0)	95 (31.8)	
Number of hours in front of the TV per day	None	2 (2.7)	18 (6.10)	0.50
	< 2	42 (56.8)	158 (53.2)	
	≥ 2	30 (40.5)	121 (40.7)	
Number of hours using internet for non-school related issues	None	8 (10.8)	62 (21.0)	0.10
	< 2	42 (56.8)	136 (46)	
	≥ 2	24 (32.4)	98 (33.1)	
Number of hours using video games	None	31 (41.9)	138 (45.9)	0.82
	< 2	31 (41.9)	119 (39.5)	
	≥ 2	12 (16.2)	44 (14.6)	
Number of hours using the mobile phone per day	None	15 (25.9)	44 (18.0)	0.40
	< 2	36 (62.1)	167 (68.4)	
	≥ 2	7 (12.1)	33 (13.5)	
Score for electronics*	0	26 (34.2)	113 (37.2)	0.88
	1	32 (42.1)	113 (37.2)	
	2	13 (17.1)	54 (17.8)	
	3+	5 (6.6)	24 (7.9)	
**Substance abuse**				
Ever smoked a cigarette		17 (23.0)	57 (18.9)	0.43
Ever tried to quit smoking in the past year		13 (92.9)	31 (75.6)	0.25
Ever smoked shisha		8 (10.8)	29 (9.6)	0.76
Ever tried to quit shisha in the past year		4 (66.7)	6 (24.0)	0.07
Ever tried sniffing (30 days )		11 (15.1)	49 (16.3)	0.79
Ever used marijuana		3 (4.2)	4 (1.3)	0.13
Ever drank alcohol		1 (1.4)	4 (1.4)	1.00
Ever used any illegal pills		2 (2.9)	5 (1.7)	0.62
Score for substance use**	0	48 (63.2)	201 (66.1)	0.87
	1	17 (22.4)	69 (22.7)	
	2	8 (10.5)	26 (8.6)	
	3+	3 (4.0)	8 (2.6)	

* Original score created by adding up the number of the 4 following suboptimal behaviors: spending ≥ 2 hours/day on TV, ≥ 2hours/day on the internet for non-school related issues, ≥ 2 hours/day on video games, or ≥ 2 hours/day on the mobile phone (a score of 1 indicates at least 2 hours on any of the 4 behaviors, a score of 2 indicates at least 4 hours, and a score of 3+ indicates at least 6 hours)

** Original score created by adding up the number of the 6 following suboptimal behaviors: any use of cigarette, shisha, alcohol, marijuana, sniffing, and illegal pills (a score of 1 indicates ever used any of the above 6 mentioned behaviors, a score of 2 indicates 2 behaviors, and a score of 3+ indicates at least 3 behaviors)

Table 5 presents results of the multivariate analysis. The only significantly associated lifestyle pattern was related to sleeping pattern where adolescents with diabetes slept longer than 10 hours during weekdays (OR = 2.35, 95% CI 1.04–5.31).

**Table 5:** Multivariate analysis of lifestyle factors and their association with diabetes status (compared to no diabetes).

		Odds Ratio	95% CI	p-value
Eating behavior score		0.83	(0.43–1.58)	0.57
**Physical activity for 30 minutes**				
	More than twice per week	REF	REF	REF
	Never	0.53	(0.28–0.99)	0.12
	1–2 times per week	0.69	(0.34–1.37)	0.86
**Number of hours slept during the weekend (hours)**				
	< 8	REF	REF	REF
	8–10	0.62	(0.33–1.18)	0.009
	> 10 hours	2.35	(1.04–5.31)	0.008
**BMI (kg/m2)**		0.99	(0.95–1.04)	0.78
**Substance abuse score**				
	None	REF	REF	REF
	One	1.13	(0.58–2.19)	0.70
	Two	1.39	(0.56–3.48)	0.80
	> Three	1.64	(0.38–7.06)	0.64
**Electronics use score**				
	None	REF	REF	REF
	One activity versus none	1.36	(0.72–2.55)	0.17
	Two activities versus none	0.96	(0.43–2.19)	0.92
	Three or more activities versus none	0.74	(0.24–2.26)	0.46

REF: Reference group to which the comparison was made.

## Discussion

This case-control nationally representative study found that overall adolescents, aged between 11 and 18 years, with diabetes in KSA have lifestyle habits which tend to be healthier than their peers with respect to some behaviors, such as eating behavior or sleeping pattern, but also engaged similarly in activities which were unhealthy such as tobacco/substance use and excessive leisurely digital screen use. They also felt sad more often but sought help for it more frequently.

Overall, the prevalence of diabetes using self-report questionnaire was 0.7% (95% CI: 0.5–0.8), among these adolescent boys and girls. This prevalence is higher than previously reported studies [21–23]: as an example, a Saudi nationwide survey conducted between 2007 and 2009 on a representative sample of 87,417 participants found the prevalence of diabetes of 0.45% (0.38% T1D and 0.07% T2D), based on self-report, in those aged ≤18 years [21]. They reported an increase in the age-specific prevalence in those with T1D and a flat rate in those with T2D. Since our sample was restricted to adolescents, the higher age range of our sample, may partially explain the higher prevalence found in the current study. Indeed, in a nationwide study conducted in KSA between 2001 and 2007 by Al-Herbish et al, and also based on self-report, the prevalence of T1D was 109 per 100,000 children (0.11%) aged ≤18 years, but the highest prevalence was found among those aged 13–16 years with 243 per 100,000 adolescents (0.24%) [22]. The prevalence in our study was also close to the NHANES 2005–14 cohort of adolescents aged 12–19 years which found a prevalence of 0.8%. However, the latter was based on self-report, as well as laboratory measurement of glucose and HbA1C [24], and included an additional 28% of participants who were not previously diagnosed. Thus, the true prevalence in our study remains higher, in absolute numbers, than the previously reported studies. It is possible that self-report in our study has overestimated the prevalence, since there was no data on medication use or medical evaluation.

Interestingly, in the current study, there was a predominance of males with diabetes (65.8%), while the original Jeeluna cohort had an equal distribution of males and females, as would be expected from a nationally representative study [17]. This is also reflected in the NHANES cohort where the gender-specific prevalence was 0.97% and 0.61% in adolescent boys and girls, respectively [24]. It is similarly reflected by a review paper from Sweden where the male prevalence ranged from 0.45 to 0.78% and the female prevalence from 0.37 to 0.44%, respectively [25]. Therefore, the current male predominance in diabetes is consistent with previous reports, and with the fact that T1D is the only common autoimmune condition where the gender predisposition risk ratio is reversed. One possible explanation is that estrogen has anti-inflammatory properties, somewhat attenuating the insulitis which occurs with autoimmune diabetes [26].

The metabolic control, in line with the definition, was worse among diabetic teens than the controls with respect to glucose and lipids. There was also a tendency towards feeling sad and down more often. The link with depression was corroborated by another study from Jeeluna which found twice the likelihood of reporting feeling sad in the presence of chronic illness [20]. Despite these findings, with respect to lifestyle habits, adolescents with diabetes had a more healthful sleep habit associated and were more often on a diet. Other health habits did not differ significantly between the two groups, such as exercise frequency or smoking/quitting smoking, possibly because both groups had overall low levels of healthful behavior. Nonetheless, those with diabetes had more awareness regarding health and more access to healthcare. Health awareness findings are supported by a cross-sectional survey of 4333 adolescent boys and girls from Kuwait public and private schools, where 67.5% of the subgroup with diabetes had health knowledge about diabetes [27].

In the current study, more health access was supported by the fact that adolescents with diabetes obtained their medical information from a healthcare provider and from family, rather than from school. They also tended to seek more help when feeling down and were more frequently on a diet. Although mere associations, these factors support the notion that the group with diabetes had more access to health care and seemed to be more health aware. The increased health awareness was similarly alluded to in a national study from Germany, the KiGGS study, which also found certain behaviors to be healthier among adolescents with T1D as compared to the general population [18].

With respect to nutrition, both groups fell short of the WHO recommendation, with only about a quarter meeting the target of 5 servings per day. This finding was consistent with adolescents from the Eastern Mediterranean region (EMRO), as described by the Global School-based Student Health survey (GSSHS) which included 11 countries from EMRO, where only 19% of adolescents met the requirements for fruit and vegetable intake. The study did not comprise KSA, however it did include other affluent countries from the Gulf region, which had similarly low fruit and vegetable intake [16, 28]. The poor nutrition in adolescents is not limited to EMRO, but is similarly found across the globe, especially in low to middle income countries, supporting the notion of nutrition transition observed over the past 30 years [29, 30].

Regarding sleep, more than half of all adolescents slept less than the required minimum of 8 hours, and this proportion decreased for both groups on weekends, but the difference was less marked for those with diabetes, alluding to the fact that the sleep debt may be less pronounced in them. The sleep debt was described in the whole original Jeeluna cohort, further supporting the fact that the diabetic group distinguished itself by having less variability in pattern between weekdays and weekends [31]. A sleep debt is considered unhealthy in general, and is reflected by variability in sleep duration between weekdays and weekends [32]. A sleep debt cannot be made up for by sleeping in on the weekends with respect to metabolic disturbances, supporting the notion that the sleep pattern observed in our group with diabetes is more healthful [33]. The finding in our study is supported objectively by a case-control study among adolescents with T1D where sleep was assessed wearing a wristwatch with an actigraph, and which found that sleep duration is on average only 45 minutes longer on week-ends than on weekdays, which is within the accepted variability of sleep [34]. In our study, adolescents with diabetes had a lower sleep debt, possibly reflecting healthier sleep habits.

The low level of physical activity among both groups was similar to the finding of the KiGGS study, where 6.4% of those with T1D and 10.2% of the reference population reported no exercise at all [18].

Similarly, with respect to electronics use, at least two thirds of both groups of adolescents spent more than two hours daily on non-scholastic screen time activity, against the recommended guidelines [9]. Furthermore, those with diabetes tended to spend more time on the internet and did more chatting. The higher use of internet among diabetic adolescents was similarly found in the KiGGS cohort, where more than 2 hours daily were spent by 16% versus 7% of cases and controls, respectively. In a systematic review assessing health-related internet usage among children and adolescents, it was found that the web is sought for information regarding chronic conditions and sensitive topics [35]. The chronicity of diabetes mellitus, in which every aspect of life may affect metabolic control, may increase the time spent on digital media related to the care of this condition [36]. It is also possible that diabetic adolescents prefer an indoor, quiet activity when not exercising.

Being an exploratory period, risk-taking behaviors may begin in adolescence. In our study, about one in every three adolescents had experimented with at least one form of substance. The risks were seen across both groups. The most common form was smoking cigarettes and shisha. However, adolescents with diabetes reported trying to quit more often than their peers, possibly reflecting more awareness. In line with this, German adolescents with diabetes were half as likely to smoke cigarettes as their peers, 10.6 versus 20.4%, respectively [18]. Both the high screen time and the substance use need further scrutiny in this age group as they may have common determinants [37].

Regardless of the diabetes status, the lifestyle of this nationally representative sample is unhealthy with a minority of adolescents meeting recommended guidelines. Our group had twice the rate of overweight/obesity than the KiGGS cohort and was five times more likely to be sedentary [18]. In a study comparing 2,806 British and Saudi adolescents for their health behavior, the prevalence of overweight/obesity was higher among the Saudi boys and girls as compared to British ones (38.3 versus 24.1%, respectively) [13]. They also had much lower rates of physical activity and energy expenditure for both genders. Interestingly, both Saudi and British participants had low rates of vegetable and fruit consumption and high screen time with 88 and 90% of Saudi and British, respectively, spending more than two hours daily. This unhealthy lifestyle is prevalent across all Arab youth surveyed [28]. Similarly, physical inactivity was strikingly prevalent in the same GSSHS cohort varying from 65 to 90% [38]. The prevalence of tobacco use, especially among boys, varied across these countries from 8 to 24% [16] and was higher than among other youth surveyed, for example in Germany [18]. Therefore, some of the unhealthy lifestyle indicators such as low fresh food intake and high screen time seem common to the current generation, and others seem more specific to Arab youth. Within this unfavorable context, the presence of diabetes seems to shift the balance towards a relatively healthier lifestyle.

Adolescence is a critical time of development, where brain maturation takes place and lifelong health habits are acquired. It represents a vulnerable time when youth may be influenced by peers, by the web, and by the ambient environment. Rather than being a simple link between childhood and adulthood, it represents an ideal opportunity for investing in health [39].

A survey of 426 Saudi adolescent boys and girls without diabetes showed that they do not feel susceptible to developing diabetes, nor do they rank T2D high on the disease severity scale [40]. Furthermore, perceived obstacles to lack of exercise were lack of time and the hot weather.

On the other hand, we have shown that despite the overall poor lifestyle habits and findings, adolescents with diabetes tend to be more healthful in their attitude and in most behaviors. In particular, behaviors reflecting better access to healthcare (such as more frequent and longer doctor’s visit, and provision of health information) were more favorable among the diabetic youth. Therefore, diabetes during adolescence should be viewed as an opportunity to instill and reinforce health habits which may last a lifetime.

This motivation should be widened and replicated across all youth. Health can be promoted by increasing awareness, by improving access to health education, and by creating a proper set up and environment for physical activity.

The relevance of this study lies in filling the gap in the availability of data about lifestyle in youth, especially in those with diabetes. This study was conducted in 2011–2012 academic year, and over the past decade, many sociodemographic and economic changes have occurred in the Arab world that have been further accelerated by the Covid-19 pandemic with lockdown and remote schooling. Nevertheless, this study on top of filling the gap mentioned earlier, can be used as a reference for any future comparisons. This study has several limitations. Firstly, the original survey was collected via a self-filled questionnaire (except for anthropometrics and lab results) and is subjective in nature, for example, in the definition of cases and controls. Moreover, the type, duration, and use of medications for diabetes were not considered, all important factors in determining health behavior. Thirdly, the calculated scores for the behaviors are not based on validated scores in the literature, however, some of their components are. We think using these scores adds a holistic capture to the individual behavior components. Finally, the study being cross-sectional in nature, cannot determine any causality and has only identified associated risks and behaviors among adolescents with diabetes. Despite the limitations, one of the strengths of this study is being a nationally representative sample, analyzed in a case-control manner. Even if subjective, the participants’ responses reflect real life attitudes and behavior. Furthermore, the MENA region, despite its heterogeneity, carries certain elements of commonality [16] making the study of interest at large. Therefore, our study adds important information to the paucity of existing data comparing lifestyle between adolescents with diabetes and those without.

## Conclusions

The current study reports a prevalence of diabetes among Saudi adolescents of 0.7%. Youth with diabetes remain far from guideline targets but seem predisposed to better lifestyle as compared to their non-diabetic peers. They have more access to health care which is a favorable element to be built upon. More prospective studies looking into health awareness and attitude, and further dissecting the obstacles to implementation of a healthy lifestyle need to be conducted.

## Supporting information

S1 Data (XLSX)

## References

[1] Mayer-Davis EJ, Lawrence JM, Dabelea D, Divers J, Isom S, Dolan L, et al. Incidence Trends of Type 1 and Type 2 Diabetes among Youths, 2002–2012. The New England journal of medicine. 2017;376(15):1419–29. doi: 10.1056/NEJMoa1610187 28402773 PMC5592722

[2] American Diabetes Association. Children and Adolescents: Standards of Medical Care in Diabetes-2019. Diabetes care. 2019;42(Suppl 1):S148–s64. doi: 10.2337/dc19-S013 30559239

[3] Quirk H, Blake H, Tennyson R, Randell TL, Glazebrook C. Physical activity interventions in children and young people with Type 1 diabetes mellitus: a systematic review with meta-analysis. Diabetic medicine: a journal of the British Diabetic Association. 2014;31(10):1163–73. doi: 10.1111/dme.12531 24965376 PMC4232875

[4] Daneman D. Early diabetes-related complications in adolescents: risk factors and screening. Hormone research. 2005;63(2):75–85. doi: 10.1159/000083692 15677872

[5] TODAY Study Group, Zeitler P, Hirst K, Pyle L, Linder B, Copeland K, et al. A clinical trial to maintain glycemic control in youth with type 2 diabetes. The New England journal of medicine. 2012;366(24):2247–56. doi: 10.1056/NEJMoa1109333 22540912 PMC3478667

[6] World Health Organization (WHO). Diet, Nutrition and the Prevention of Chronic Diseases. Healthy diet October 2018 [Available from: https://www.who.int/en/news-room/fact-sheets/detail/healthy-diet

[7] Wisting L, Reas DL, Bang L, Skrivarhaug T, Dahl-Jorgensen K, Ro O. Eating patterns in adolescents with type 1 diabetes: Associations with metabolic control, insulin omission, and eating disorder pathology. Appetite. 2017;114:226–31. doi: 10.1016/j.appet.2017.03.035 28351671

[8] Paruthi S, Brooks LJ, D’Ambrosio C, Hall WA, Kotagal S, Lloyd RM, et al. Recommended Amount of Sleep for Pediatric Populations: A Consensus Statement of the American Academy of Sleep Medicine. Journal of clinical sleep medicine: JCSM: official publication of the American Academy of Sleep Medicine. 2016;12(6):785–6. doi: 10.5664/jcsm.5866 27250809 PMC4877308

[9] American Academy of Pediatrics (AAP) POLICY STATEMENT. Children, Adolescents, and the Media 2013 [updated October 31, 2018. Available from: www.aappublications.org/news.

[10] Galler A, Lindau M, Ernert A, Thalemann R, Raile K. Associations between media consumption habits, physical activity, socioeconomic status, and glycemic control in children, adolescents, and young adults with type 1 diabetes. Diabetes care. 2011;34(11):2356–9. doi: 10.2337/dc11-0838 21926289 PMC3198300

[11] Rausch JR, Hood KK, Delamater A, Shroff Pendley J, Rohan JM, Reeves G, et al. Changes in treatment adherence and glycemic control during the transition to adolescence in type 1 diabetes. Diabetes care. 2012;35(6):1219–24. doi: 10.2337/dc11-2163 22474040 PMC3357213

[12] Borus JS, Laffel L. Adherence challenges in the management of type 1 diabetes in adolescents: prevention and intervention. Current opinion in pediatrics. 2010;22(4):405–11. doi: 10.1097/MOP.0b013e32833a46a7 20489639 PMC3159529

[13] Al-Hazzaa HM, Al-Nakeeb Y, Duncan MJ, Al-Sobayel HI, Abahussain NA, Musaiger AO, et al. A cross-cultural comparison of health behaviors between Saudi and British adolescents living in urban areas: gender by country analyses. International journal of environmental research and public health. 2013;10(12):6701–20. doi: 10.3390/ijerph10126701 24300072 PMC3881136

[14] Abuyassin B, Laher I. Diabetes epidemic sweeping the Arab world. World journal of diabetes. 2016;7(8):165–74. doi: 10.4239/wjd.v7.i8.165 27114755 PMC4835661

[15] Asirvatham A, Al-Dawish A, Mujammami M, Dawish MAA. Type 1 Diabetes Mellitus in Saudi Arabia: A Soaring Epidemic. International journal of pediatrics. 2018;2018:9408370. doi: 10.1155/2018/9408370 29853923 PMC5964576

[16] Obermeyer C. Adolescents in Arabic countries: health statistics and social context. DIFI Family Research and Proceedings. 2015(1).

[17] AlBuhairan FS, Tamim H, Al Dubayee M, AlDhukair S, Al Shehri S, Tamimi W, et al. Time for an Adolescent Health Surveillance System in Saudi Arabia: Findings From "Jeeluna". The Journal of adolescent health: official publication of the Society for Adolescent Medicine. 2015;57(3):263–9. doi: 10.1016/j.jadohealth.2015.06.009 26299553

[18] Kummer S, Stahl-Pehe A, Castillo K, Bachle C, Graf C, Strassburger K, et al. Health behaviour in children and adolescents with type 1 diabetes compared to a representative reference population. PloS one. 2014;9(11):e112083. doi: 10.1371/journal.pone.0112083 25384048 PMC4226508

[19] CDC. Defining childhood obesity [Internet]. 2018 [updated 2018 July Available from: https://www.cdc.gov/obesity/childhood/defining.html.

[20] Abou Abbas O, AlBuhairan F. Predictors of adolescents’ mental health problems in Saudi Arabia: findings from the Jeeluna® national study. Child and adolescent psychiatry and mental health. 2017;11:52. doi: 10.1186/s13034-017-0188-x 28959356 PMC5615485

[21] Al-Rubeaan K. National surveillance for type 1, type 2 diabetes and prediabetes among children and adolescents: a population-based study (SAUDI-DM). Journal of epidemiology and community health. 2015;69(11):1045–51. doi: 10.1136/jech-2015-205710 26085648 PMC4680138

[22] Al-Herbish AS, El-Mouzan MI, Al-Salloum AA, Al-Qurachi MM, Al-Omar AA. Prevalence of type 1 diabetes mellitus in Saudi Arabian children and adolescents. Saudi medical journal. 2008;29(9):1285–8. 18813413

[23] Patterson C, Guariguata L, Dahlquist G, Soltesz G, Ogle G, Silink M. Diabetes in the young—a global view and worldwide estimates of numbers of children with type 1 diabetes. Diabetes research and clinical practice. 2014;103(2):161–75. doi: 10.1016/j.diabres.2013.11.005 24331235

[24] Menke A, Casagrande S, Cowie CC. Prevalence of Diabetes in Adolescents Aged 12 to 19 Years in the United States, 2005–2014. Jama. 2016;316(3):344–5. doi: 10.1001/jama.2016.8544 27434447

[25] Wandell PE, Carlsson AC. Time trends and gender differences in incidence and prevalence of type 1 diabetes in Sweden. Current diabetes reviews. 2013;9(4):342–9. doi: 10.2174/15733998113099990064 23721159

[26] Mauvais-Jarvis F. Gender differences in glucose homeostasis and diabetes. Physiology & behavior. 2018;187:20–3. doi: 10.1016/j.physbeh.2017.08.016 28843891 PMC5826763

[27] Al-Hussaini M, Mustafa S. Adolescents’ knowledge and awareness of diabetes mellitus in Kuwait. Alexandria Journal of Medicine [Internet]. 2016 September 2018; 52(1):[61–6 pp.]. Available from: 10.1016/j.ajme.2015.04.001.

[28] Al Ani MF, Al Subhi LK, Bose S. Consumption of fruits and vegetables among adolescents: a multi-national comparison of eleven countries in the Eastern Mediterranean Region. The British journal of nutrition. 2016;115(6):1092–9. doi: 10.1017/S0007114515005371 26817392

[29] Mehio Sibai A, Nasreddine L, Mokdad AH, Adra N, Tabet M, Hwalla N. Nutrition transition and cardiovascular disease risk factors in Middle East and North Africa countries: reviewing the evidence. Annals of nutrition & metabolism. 2010;57(3–4):193–203. doi: 10.1159/000321527 21088386

[30] Popkin BM. Nutrition Transition and the Global Diabetes Epidemic. Current diabetes reports. 2015;15(9):64. doi: 10.1007/s11892-015-0631-4 26209940 PMC4942180

[31] Nasim M, Saade M, AlBuhairan F. Sleep deprivation: prevalence and associated factors among adolescents in Saudi Arabia. Sleep medicine. 2019;53:165–71. doi: 10.1016/j.sleep.2018.08.031 30529632

[32] Owens J. Insufficient sleep in adolescents and young adults: an update on causes and consequences. Pediatrics. 2014;134(3):e921–32. doi: 10.1542/peds.2014-1696 25157012 PMC8194472

[33] Depner CM, Melanson EL, Eckel RH, Snell-Bergeon JK, Perreault L, Bergman BC, et al. Ad libitum Weekend Recover Sleep Fails to Prevent Metabolic Dysregulation during a Repeating Pattern of Insufficient Sleep and Weekend Recovery Sleep. Current biology: CB. 2019;29(6):957–67.e4. doi: 10.1016/j.cub.2019.01.069 30827911 PMC12798825

[34] Patel NJ, Savin KL, Kahanda SN, Malow BA, Williams LA, Lochbihler G, et al. Sleep habits in adolescents with type 1 diabetes: Variability in sleep duration linked with glycemic control. Pediatric diabetes. 2018. doi: 10.1111/pedi.12689 29708297 PMC6207484

[35] Park E, Kwon M. Health-Related Internet Use by Children and Adolescents: Systematic Review. Journal of medical Internet research. 2018;20(4):e120. doi: 10.2196/jmir.7731 29615385 PMC5904452

[36] Vaala SE, Hood KK, Laffel L, Kumah-Crystal YA, Lybarger CK, Mulvaney SA. Use of Commonly Available Technologies for Diabetes Information and Self-Management Among Adolescents With Type 1 Diabetes and Their Parents: A Web-Based Survey Study. Interactive journal of medical research. 2015;4(4):e24. doi: 10.2196/ijmr.4504 26715191 PMC4710846

[37] AlSayyari A, AlBuhairan F. Relationship of media exposure to substance use among adolescents in Saudi Arabia: Results from a national study. Drug and alcohol dependence. 2018;191:174–80. doi: 10.1016/j.drugalcdep.2018.01.025 30121476

[38] Sharara E, Akik C, Ghattas H, Makhlouf Obermeyer C. Physical inactivity, gender and culture in Arab countries: a systematic assessment of the literature. BMC public health. 2018;18(1):639. doi: 10.1186/s12889-018-5472-z 29776343 PMC5960209

[39] AlBuhairan FS. Adolescent and Young Adult Health in the Arab Region: Where We Are and What We Must Do (Editorial). J Adol Health 2015; 57: 249–251. 10.1016/j.jadohealth.2015.06.010 26299552

[40] Al-Mutairi RL, Amen A Bawazir AA, Ahmed AE, Jradi H. Health Beliefs Related to Diabetes Mellitus Prevention among Adolescents in Saudi Arabia. Sultan Qaboos Univ Med J 2015;15(3):e398–408. doi: 10.18295/squmj.2015.15.03.015 26355752 PMC4554276

